# 3-Cyclo­propyl-1-(4-methyl­phenyl­sulfon­yl)piperidine-3,5-diol

**DOI:** 10.1107/S1600536811054420

**Published:** 2011-12-23

**Authors:** Yi Wang, Yong-Yue Lin

**Affiliations:** a College of Chemistry and Engineering, Yunnan Normal University, Kunming, People’s Republic of China

## Abstract

In the title compound, C_15_H_21_NO_4_S, both hy­droxy groups on the piperidine ring are located in axial positions, whereas the tosyl group and the cyclo­propane ring are in equatorial positions. An intra­molecular O—H⋯O hydrogen bond occurs. In the crystal, mol­ecules form inversion dimers *via* pairs of O—H⋯O hydrogen bonds, generating cyclic *R*_4_^4^(8) motifs, as noted previously in related diols.

## Related literature

Aza­cyclo­hexa­nediol (piperidine­diol) derivatives are widely found in natural products and are often incorporated into drugs, see: Nagahama *et al.* (2003 ▶); Fukushima *et al.* (2001 ▶). For related structures, see: Hidekazu *et al.* (2005 ▶); Karin *et al.* (2006 ▶). Similar hydrogen bonding has been seen in related diols, see: Ferguson *et al.* (1993 ▶).
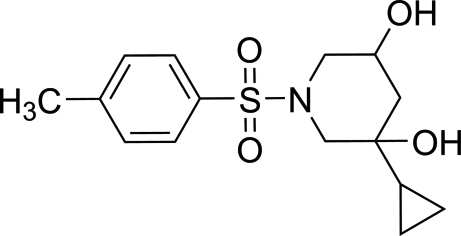


## Experimental

### 

#### Crystal data

C_15_H_21_NO_4_S
*M* *_r_* = 311.39
Monoclinic, 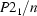

*a* = 11.583 (5) Å
*b* = 5.598 (2) Å
*c* = 24.009 (9) Å
β = 102.905 (7)°
*V* = 1517.5 (10) Å^3^
*Z* = 4
Mo *K*α radiation
μ = 0.23 mm^−1^
*T* = 173 K
0.24 × 0.11 × 0.06 mm

#### Data collection

Rigaku MM007-HF CCD (Saturn 724+) diffractometer
Absorption correction: numerical (*CrystalClear*; Rigaku, 2002 ▶) *T*_min_ = 0.947, *T*_max_ = 0.986
9795 measured reflections
2676 independent reflections
2431 reflections with *I* > 2σ(*I*)
*R*_int_ = 0.053

#### Refinement

*R*[*F* ^2^ > 2σ(*F* ^2^)] = 0.069
*wR*(*F* ^2^) = 0.128
*S* = 1.23
2676 reflections
191 parameters
H-atom parameters constrained
Δρ_max_ = 0.42 e Å^−3^
Δρ_min_ = −0.32 e Å^−3^

### 

Data collection: *CrystalClear* (Rigaku, 2007 ▶); cell refinement: *CrystalClear*; data reduction: *CrystalClear*; program(s) used to solve structure: *SHELXS97* (Sheldrick, 2008 ▶); program(s) used to refine structure: *SHELXL97* (Sheldrick, 2008 ▶); molecular graphics: *DIAMOND* (Brandenburg, 1999 ▶); software used to prepare material for publication: *SHELXL97*.

## Supplementary Material

Crystal structure: contains datablock(s) I, global. DOI: 10.1107/S1600536811054420/gg2064sup1.cif

Structure factors: contains datablock(s) I. DOI: 10.1107/S1600536811054420/gg2064Isup2.hkl

Supplementary material file. DOI: 10.1107/S1600536811054420/gg2064Isup3.cml

Additional supplementary materials:  crystallographic information; 3D view; checkCIF report

## Figures and Tables

**Table 1:** Hydrogen-bond geometry (Å, °)

*D*—H⋯*A*	*D*—H	H⋯*A*	*D*⋯*A*	*D*—H⋯*A*
O3—H3*B*⋯O4	0.84	2.08	2.804 (3)	145
O4—H4⋯O3^i^	0.84	2.01	2.837 (3)	167

Symmetry code: (i) 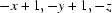.

**Fig. 1.**
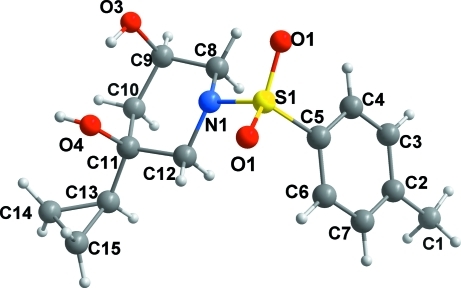
The molecular structure of the title compound, with atom labels and 30% probability displacement ellipsoids for non-H atoms.

**Fig. 2.**
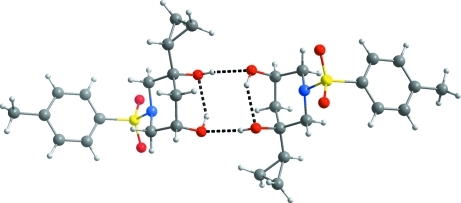
The dimeric unit showing the hydrogen bonding as dashed lines.

## supplementary crystallographic information

### Comment

Azacyclohexanediol (piperidinediol) derivatives are widely found in natural products and often incorporated into drugs (Nagahama *et al.*, 2003). We report here the crystal structure of the title compound, a 1,3-piperidinediol derivative.

Bond lengths and angles were normal. The distance of N1—C8 and N1—C12 are 1.471 (4) and 1.478 (4) Å, respectively, which are in good agreement with normal N—C bond lengths [reported values range from 1.33 to 1.52 Å]. As shown as in Fig. 1, the two hydroxy groups in piperidine ring adopt a *cis* conformation. The twist angles of hydroxy groups and the piperidine are 112.0 (3) and 110.3 (3), respectively. Thus, the orientation of both hydroxy groups are axial in reference to the piperidine ring. A comparison with analogous 1,3-cyclohexanediol systems, shows that our observed system is in good agreement with similar 1,3-cyclohexanediols (Hidekazu *et al.*, 2005; Karin *et al.*, 2006). The cyclopropane C13—C15 planes is nearly coplanar with the azacyclohexane ring. A centrosymmetric dimer is generated by intermolecular O—H—O hydrogen bonding, and this has been seen previously in related diols (Ferguson *et al.*, 1993), as shown in Fig. 2.

### Experimental

A mixture of *N*-(2-cyclopropylallyl)-4-methyl-N–(2-oxoethyl)benzenesulfonamide (1 mmol), ferric chloride hexahydrate (0.1mmol) was stirred in dichloromethane (5 ml) for 24 h. The mixture was concentrated and the residue was purified by flash column chromatography with silica gel to afford the title products (yield 55%). Single crystals suitable for X-ray diffraction were grown by slow diffusion of ether into a solution of the compound in dichloromethane.

### Refinement

All H atoms were placed in calculated positions, with C—H = 0.93—0.98 Å, and refined using a riding model, with *U*_iso_(H) = 1.2*U*_eq_(C).

### Crystal data

**Table:**

C_15_H_21_NO_4_S	*F*(000) = 664
*M**_r_* = 311.39	*D*_x_ = 1.363 Mg m^−^^3^
Monoclinic, *P*2_1_/*n*	Mo *K*α radiation, λ = 0.71073 Å
Hall symbol: -p 2yn	Cell parameters from 345 reflections
*a* = 11.583 (5) Å	θ = 2.2–27.5°
*b* = 5.598 (2) Å	µ = 0.23 mm^−^^1^
*c* = 24.009 (9) Å	*T* = 173 K
β = 102.905 (7)°	Plate, colorless
*V* = 1517.5 (10) Å^3^	0.24 × 0.11 × 0.06 mm
*Z* = 4	

### Data collection

**Table:**

Rigaku MM007-HF CCD (Saturn 724+) diffractometer	2676 independent reflections
Radiation source: rotating anode	2431 reflections with *I* > 2σ(*I*)
Confocal	*R*_int_ = 0.053
ω scans at fixed χ = 45°	θ_max_ = 25.0°, θ_min_ = 1.7°
Absorption correction: numerical (*CrystalClear*; Rigaku, 2002)	*h* = −13→13
*T*_min_ = 0.947, *T*_max_ = 0.986	*k* = −6→6
9795 measured reflections	*l* = −28→25

### Refinement

**Table:**

Refinement on *F*^2^	Primary atom site location: structure-invariant direct methods
Least-squares matrix: full	Secondary atom site location: difference Fourier map
*R*[*F*^2^ > 2σ(*F*^2^)] = 0.069	Hydrogen site location: inferred from neighbouring sites
*wR*(*F*^2^) = 0.128	H-atom parameters constrained
*S* = 1.23	*w* = 1/[σ^2^(*F*_o_^2^) + (0.0211*P*)^2^ + 2.2306*P*] where *P* = (*F*_o_^2^ + 2*F*_c_^2^)/3
2676 reflections	(Δ/σ)_max_ < 0.001
191 parameters	Δρ_max_ = 0.42 e Å^−^^3^
0 restraints	Δρ_min_ = −0.32 e Å^−^^3^

### Fractional atomic coordinates and isotropic or equivalent isotropic displacement parameters (Å^2^)

**Table:**

	*x*	*y*	*z*	*U*_iso_*/*U*_eq_	
S1	0.48729 (7)	0.71212 (14)	0.21603 (3)	0.0254 (2)	
O1	0.3693 (2)	0.8072 (4)	0.20391 (9)	0.0309 (6)	
O2	0.5867 (2)	0.8711 (4)	0.22413 (9)	0.0333 (6)	
O3	0.6103 (2)	0.5720 (4)	0.06890 (9)	0.0323 (6)	
H3B	0.5388	0.5980	0.0535	0.049*	
O4	0.3664 (2)	0.4775 (4)	0.04568 (9)	0.0293 (5)	
H4	0.3722	0.4391	0.0126	0.044*	
N1	0.4962 (2)	0.5392 (5)	0.16189 (10)	0.0239 (6)	
C1	0.5488 (4)	0.0129 (7)	0.41161 (16)	0.0499 (11)	
H1A	0.5012	0.0572	0.4390	0.075*	
H1B	0.6325	0.0049	0.4312	0.075*	
H1C	0.5231	−0.1434	0.3950	0.075*	
C2	0.5327 (3)	0.1979 (6)	0.36466 (13)	0.0336 (8)	
C3	0.6307 (3)	0.3092 (6)	0.35169 (13)	0.0314 (8)	
H3A	0.7078	0.2742	0.3736	0.038*	
C4	0.6166 (3)	0.4712 (6)	0.30688 (13)	0.0291 (8)	
H4A	0.6839	0.5447	0.2976	0.035*	
C5	0.5040 (3)	0.5246 (6)	0.27584 (12)	0.0242 (7)	
C6	0.4056 (3)	0.4195 (6)	0.28916 (14)	0.0331 (8)	
H6A	0.3282	0.4584	0.2682	0.040*	
C7	0.4216 (3)	0.2568 (7)	0.33361 (14)	0.0390 (9)	
H7A	0.3543	0.1840	0.3429	0.047*	
C8	0.6136 (3)	0.4325 (6)	0.16365 (13)	0.0284 (8)	
H8A	0.6255	0.2908	0.1890	0.034*	
H8B	0.6768	0.5498	0.1788	0.034*	
C9	0.6192 (3)	0.3603 (6)	0.10331 (13)	0.0296 (8)	
H9A	0.6973	0.2823	0.1043	0.036*	
C10	0.5208 (3)	0.1832 (6)	0.07993 (14)	0.0298 (8)	
H10A	0.5367	0.0323	0.1018	0.036*	
H10B	0.5213	0.1474	0.0396	0.036*	
C11	0.3986 (3)	0.2754 (6)	0.08316 (13)	0.0262 (7)	
C12	0.4001 (3)	0.3648 (6)	0.14376 (12)	0.0255 (7)	
H12A	0.3231	0.4402	0.1445	0.031*	
H12B	0.4118	0.2281	0.1706	0.031*	
C13	0.3070 (3)	0.0790 (6)	0.06840 (13)	0.0308 (8)	
H13A	0.3219	−0.0615	0.0948	0.037*	
C14	0.2511 (3)	0.0174 (7)	0.00777 (14)	0.0370 (9)	
H14A	0.2363	−0.1534	−0.0019	0.044*	
H14B	0.2723	0.1158	−0.0227	0.044*	
C15	0.1790 (3)	0.1328 (7)	0.04522 (15)	0.0415 (9)	
H15A	0.1560	0.3021	0.0377	0.050*	
H15B	0.1199	0.0330	0.0585	0.050*	

### Atomic displacement parameters (Å^2^)

**Table:**

	*U*^11^	*U*^22^	*U*^33^	*U*^12^	*U*^13^	*U*^23^
S1	0.0328 (5)	0.0215 (4)	0.0217 (4)	0.0001 (4)	0.0057 (3)	−0.0003 (3)
O1	0.0350 (14)	0.0271 (13)	0.0300 (12)	0.0092 (11)	0.0062 (10)	−0.0013 (10)
O2	0.0402 (15)	0.0251 (12)	0.0354 (13)	−0.0088 (11)	0.0105 (11)	−0.0012 (10)
O3	0.0342 (14)	0.0349 (14)	0.0274 (12)	0.0003 (11)	0.0057 (10)	0.0043 (10)
O4	0.0428 (14)	0.0237 (12)	0.0214 (11)	0.0061 (11)	0.0074 (10)	0.0032 (9)
N1	0.0276 (15)	0.0247 (15)	0.0201 (13)	0.0006 (12)	0.0066 (11)	0.0001 (11)
C1	0.058 (3)	0.050 (3)	0.040 (2)	0.000 (2)	0.0071 (19)	0.0173 (19)
C2	0.039 (2)	0.038 (2)	0.0236 (17)	0.0013 (18)	0.0070 (15)	0.0045 (16)
C3	0.0287 (19)	0.040 (2)	0.0234 (16)	0.0039 (16)	0.0011 (14)	0.0009 (15)
C4	0.0287 (19)	0.0338 (19)	0.0257 (17)	−0.0059 (16)	0.0083 (14)	−0.0033 (15)
C5	0.0292 (18)	0.0250 (17)	0.0182 (15)	−0.0005 (15)	0.0050 (13)	−0.0019 (13)
C6	0.0257 (19)	0.044 (2)	0.0289 (18)	−0.0042 (17)	0.0041 (14)	0.0028 (16)
C7	0.032 (2)	0.051 (2)	0.0355 (19)	−0.0083 (19)	0.0124 (16)	0.0103 (18)
C8	0.0316 (19)	0.0290 (19)	0.0243 (16)	0.0032 (15)	0.0058 (14)	0.0022 (15)
C9	0.034 (2)	0.0289 (19)	0.0269 (17)	0.0089 (15)	0.0084 (15)	0.0039 (14)
C10	0.040 (2)	0.0239 (18)	0.0265 (17)	0.0053 (15)	0.0102 (15)	−0.0025 (14)
C11	0.0379 (19)	0.0191 (17)	0.0216 (16)	0.0025 (15)	0.0064 (14)	0.0030 (13)
C12	0.0289 (19)	0.0237 (17)	0.0238 (16)	0.0004 (14)	0.0056 (14)	0.0006 (13)
C13	0.042 (2)	0.0240 (18)	0.0244 (17)	−0.0001 (16)	0.0030 (15)	0.0018 (14)
C14	0.045 (2)	0.032 (2)	0.0307 (19)	0.0002 (18)	0.0025 (16)	−0.0053 (16)
C15	0.038 (2)	0.041 (2)	0.046 (2)	−0.0009 (18)	0.0112 (18)	−0.0024 (18)

### Geometric parameters (Å, °)

**Table:**

S1—O2	1.434 (2)	C6—H6A	0.9500
S1—O1	1.435 (2)	C7—H7A	0.9500
S1—N1	1.642 (3)	C8—C9	1.519 (4)
S1—C5	1.754 (3)	C8—H8A	0.9900
O3—C9	1.435 (4)	C8—H8B	0.9900
O3—H3B	0.8400	C9—C10	1.520 (5)
O4—C11	1.442 (4)	C9—H9A	1.0000
O4—H4	0.8401	C10—C11	1.526 (4)
N1—C12	1.471 (4)	C10—H10A	0.9900
N1—C8	1.478 (4)	C10—H10B	0.9900
C1—C2	1.511 (5)	C11—C13	1.514 (5)
C1—H1A	0.9800	C11—C12	1.535 (4)
C1—H1B	0.9800	C12—H12A	0.9900
C1—H1C	0.9800	C12—H12B	0.9900
C2—C7	1.375 (5)	C13—C15	1.493 (5)
C2—C3	1.389 (5)	C13—C14	1.495 (4)
C3—C4	1.389 (4)	C13—H13A	1.0000
C3—H3A	0.9500	C14—C15	1.502 (5)
C4—C5	1.383 (4)	C14—H14A	0.9900
C4—H4A	0.9500	C14—H14B	0.9900
C5—C6	1.382 (4)	C15—H15A	0.9900
C6—C7	1.384 (5)	C15—H15B	0.9900
			
O2—S1—O1	119.80 (14)	O3—C9—C10	112.0 (3)
O2—S1—N1	106.43 (13)	C8—C9—C10	109.8 (3)
O1—S1—N1	106.46 (13)	O3—C9—H9A	108.9
O2—S1—C5	108.78 (15)	C8—C9—H9A	108.9
O1—S1—C5	108.30 (14)	C10—C9—H9A	108.9
N1—S1—C5	106.28 (14)	C9—C10—C11	112.8 (3)
C9—O3—H3B	109.5	C9—C10—H10A	109.0
C11—O4—H4	109.0	C11—C10—H10A	109.0
C12—N1—C8	111.8 (2)	C9—C10—H10B	109.0
C12—N1—S1	116.65 (19)	C11—C10—H10B	109.0
C8—N1—S1	115.8 (2)	H10A—C10—H10B	107.8
C2—C1—H1A	109.5	O4—C11—C13	110.7 (3)
C2—C1—H1B	109.5	O4—C11—C10	110.3 (2)
H1A—C1—H1B	109.5	C13—C11—C10	110.6 (3)
C2—C1—H1C	109.5	O4—C11—C12	106.4 (2)
H1A—C1—H1C	109.5	C13—C11—C12	108.5 (3)
H1B—C1—H1C	109.5	C10—C11—C12	110.1 (3)
C7—C2—C3	118.9 (3)	N1—C12—C11	110.2 (2)
C7—C2—C1	120.9 (3)	N1—C12—H12A	109.6
C3—C2—C1	120.2 (3)	C11—C12—H12A	109.6
C4—C3—C2	120.4 (3)	N1—C12—H12B	109.6
C4—C3—H3A	119.8	C11—C12—H12B	109.6
C2—C3—H3A	119.8	H12A—C12—H12B	108.1
C5—C4—C3	119.5 (3)	C15—C13—C14	60.4 (2)
C5—C4—H4A	120.3	C15—C13—C11	121.7 (3)
C3—C4—H4A	120.3	C14—C13—C11	121.6 (3)
C6—C5—C4	120.7 (3)	C15—C13—H13A	114.2
C6—C5—S1	119.9 (2)	C14—C13—H13A	114.2
C4—C5—S1	119.3 (2)	C11—C13—H13A	114.2
C5—C6—C7	118.9 (3)	C13—C14—C15	59.8 (2)
C5—C6—H6A	120.6	C13—C14—H14A	117.8
C7—C6—H6A	120.6	C15—C14—H14A	117.8
C2—C7—C6	121.6 (3)	C13—C14—H14B	117.8
C2—C7—H7A	119.2	C15—C14—H14B	117.8
C6—C7—H7A	119.2	H14A—C14—H14B	114.9
N1—C8—C9	108.4 (3)	C13—C15—C14	59.9 (2)
N1—C8—H8A	110.0	C13—C15—H15A	117.8
C9—C8—H8A	110.0	C14—C15—H15A	117.8
N1—C8—H8B	110.0	C13—C15—H15B	117.8
C9—C8—H8B	110.0	C14—C15—H15B	117.8
H8A—C8—H8B	108.4	H15A—C15—H15B	114.9
O3—C9—C8	108.4 (3)		
			
O2—S1—N1—C12	−177.7 (2)	C12—N1—C8—C9	64.1 (3)
O1—S1—N1—C12	−48.9 (2)	S1—N1—C8—C9	−159.1 (2)
C5—S1—N1—C12	66.4 (2)	N1—C8—C9—O3	63.8 (3)
O2—S1—N1—C8	47.6 (2)	N1—C8—C9—C10	−58.8 (3)
O1—S1—N1—C8	176.4 (2)	O3—C9—C10—C11	−66.3 (3)
C5—S1—N1—C8	−68.3 (2)	C8—C9—C10—C11	54.2 (4)
C7—C2—C3—C4	−2.1 (5)	C9—C10—C11—O4	66.2 (3)
C1—C2—C3—C4	177.1 (3)	C9—C10—C11—C13	−170.9 (3)
C2—C3—C4—C5	1.2 (5)	C9—C10—C11—C12	−50.9 (3)
C3—C4—C5—C6	0.3 (5)	C8—N1—C12—C11	−61.5 (3)
C3—C4—C5—S1	−175.3 (2)	S1—N1—C12—C11	162.1 (2)
O2—S1—C5—C6	156.2 (3)	O4—C11—C12—N1	−66.4 (3)
O1—S1—C5—C6	24.5 (3)	C13—C11—C12—N1	174.4 (3)
N1—S1—C5—C6	−89.5 (3)	C10—C11—C12—N1	53.2 (3)
O2—S1—C5—C4	−28.1 (3)	O4—C11—C13—C15	−32.1 (4)
O1—S1—C5—C4	−159.8 (2)	C10—C11—C13—C15	−154.7 (3)
N1—S1—C5—C4	86.1 (3)	C12—C11—C13—C15	84.4 (4)
C4—C5—C6—C7	−1.0 (5)	O4—C11—C13—C14	40.3 (4)
S1—C5—C6—C7	174.6 (3)	C10—C11—C13—C14	−82.3 (4)
C3—C2—C7—C6	1.5 (6)	C12—C11—C13—C14	156.8 (3)
C1—C2—C7—C6	−177.7 (3)	C11—C13—C14—C15	−111.1 (4)
C5—C6—C7—C2	0.1 (5)	C11—C13—C15—C14	110.9 (3)

### Hydrogen-bond geometry (Å, °)

**Table:**

*D*—H···*A*	*D*—H	H···*A*	*D*···*A*	*D*—H···*A*
O3—H3B···O4	0.84	2.08	2.804 (3)	145.
O4—H4···O3^i^	0.84	2.01	2.837 (3)	167.

Symmetry codes: (i) −*x*+1, −*y*+1, −*z*.
